# Dermoscopy as a technique for the early identification of foot melanoma

**DOI:** 10.1186/1757-1146-2-14

**Published:** 2009-05-12

**Authors:** Ivan R Bristow, Jonathan Bowling

**Affiliations:** 1 School of Health Sciences, University of Southampton, UK; 2 Department of Dermatology, The Churchill Hospital, Oxford, UK

## Abstract

Malignant melanoma is the most common primary malignant tumour arising on the foot. Where improvements in the prognosis have been observed for patients with melanoma elsewhere on the skin, pedal lesions are still frequently delayed in presentation through neglect or misdiagnosis. Detection of foot melanoma relies on the health care practitioner's skills and observations in recognising early changes. Recent publications have documented the use a dermoscopy as a tool to improve recognition of such suspicious lesions. This paper reviews current literature with a special emphasis of its potential applications on plantar and nail unit melanoma. Data from these studies suggest that the technique is a useful and significant adjunct to clinical examination, which ultimately may lead to earlier recognition of this aggressive tumour.

## Introduction

Cancers involving the skin account for a third of all human cancers. According to the World Health Organisation, malignant melanoma (MM) accounts for an estimated 132 000 new cases annually and around 66 000 deaths. Globally the incidence of the disease continues to rise, particularly in Caucasian populations [1]. As there is no effective treatment for the disease, improving survival still remains around earlier detection of malignant lesions. The thinner the lesion at diagnosis, the better the prognosis [2]. There is some evidence to suggest that patients are presenting earlier and that the mean melanoma thickness at diagnosis is declining [3], although risk factors such as older age, male gender and low educational level still predict higher thickness at presentation [4-6].

## Melanoma and the foot

**Figure 1:**
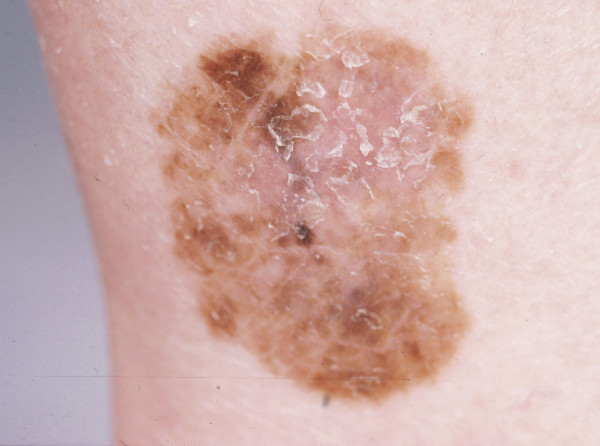
**Superficial spreading melanoma on the ankle**.

**Figure 2:**
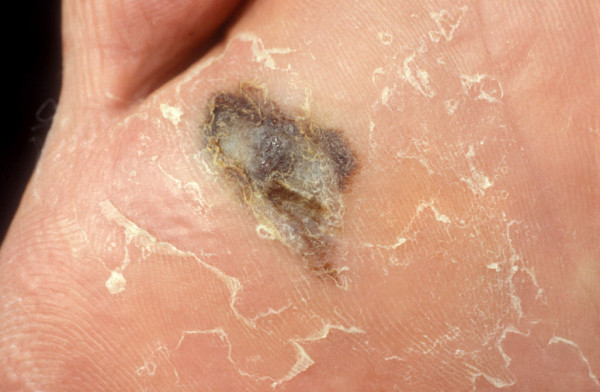
**Acral lentiginous melanoma**.

Malignant melanoma is the most common primary, malignant tumour of the foot [7] accounting for between 3–15% of all cutaneous melanoma [8]. Whereas improvements have been seen in the prognosis for some patients with melanoma, pedal lesions are still a major concern. The three most common types occurring on the foot are the superficial spreading (figure 1), nodular and acral lentiginous melanoma (ALM – figure 2). ALM is particularly prevalent on the foot as it has a predilection for the soles and nail unit [9]. In addition, it is a sub-type of melanoma that affects all skin types [10]. Day [11] identified MM on the foot as an independent risk factor for disease recurrence. This was examined further by Hsueh and colleagues [12] who reviewed 652 cases of cutaneous melanoma and analysed data comparing anatomical location to survival rates. Controlling for other variables including tumour thickness, their results confirmed that primary melanoma on the foot had a 5 year survival rate of 77% compared with 94% and 95% for lesions on the calf and thigh respectively. They concluded that the prognosis deteriorated the further the lesion was from the trunk.

From the available data, the reason for this is not clear but is probably less likely to do with the physical nature of the tumour and more to do with delays in presentation and diagnosis. Prognosis, in part, is worsened in foot melanoma as lesions frequently present later and are therefore thicker at diagnosis [13]. Reasons for patient delays have been well studied [5,14-17]. Richard et al studied 590 melanoma patients and reported a number of factors that predicted thicker lesions including melanoma which were out of the patients view (such as the plantar surface of the foot). From a medical perspective longer physician delays in diagnosis have also been observed with acral lesions [18]. Misdiagnosis could also explain a reduced prognosis in patients with acral melanoma. Bristow and Acland [19], reviewing 27 cases of acral lentiginous melanoma on the foot suggested a misdiagnosis rate of 33% whilst other workers have reported much higher rates of up to 60% in melanomas of the foot [20]. Metzger and co-workers [21] in a review of delayed diagnosis of melanoma highlighted that many acral melanoma are initially presented to non-dermatologists because patients do not suspect the problem to be a melanoma. As such clinicians are less aware of the condition; mis-diagnosis would be more of an issue. Illustrating this, many papers have been published highlighting foot melanoma misdiagnosed as other conditions such as fungal infection, onychomycosis, ulceration, haematoma and other more common foot pathologies [20,22-27].

## Detection of melanoma

The value of educating patients and practitioners through melanoma awareness campaigns cannot be emphasized too strongly and various initiatives have tried to heighten the public awareness and monitoring of skin. Equally important is the role of the practitioner in screening patients – physician detected melanomas have been shown to be significantly thinner at diagnosis than those detected by patients [6]. The ABCD rule, devised in 1985 by Freidman [28] has been well used as a mnemonic in skin assessment for recognising change in melanocytic naevi. Its value in foot melanoma has been questioned as acral lesions do not exhibit the typical features of malignant melanoma elsewhere on the skin [19,21]. Therefore at a clinical level, the decision to monitor, excise or refer on a suspicious lesion can be a difficult one.

## Dermoscopy

**Figure 3:**
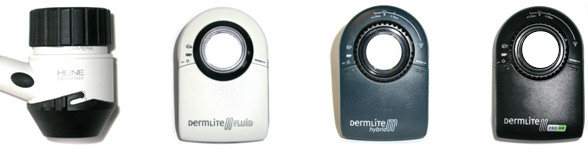
**Dermatoscopes**.

Visual examination of a suspicious skin lesion such as a melanoma can be significantly enhanced by the addition of surface microscopy. This was first recognised by Scottish Dermatologist Rona MacKie who in 1971 published a paper which demonstrated pre-operatively, the high predictive value of close examination of melanoma [29]. The difficulty arises however in that evaluation of the skin under normal conditions, with a standard magnifier, is limited due to surface reflection and refraction. To overcome this the dermatoscope is a simple, and relatively cheap, hand held magnifying device (typically 10×) which uses an oil medium or cross-polarised light allowing the viewer to observe structures deeper in the skin, not normally visible to the naked eye (figure 3). Since the 1980's the idea of "dermoscopy" began to gain momentum and its popularity as a tool aiding clinical decision making increased, particularly in Europe as more research evidence was published. In 1990, around 13 papers were published; in 2007 it had risen to over 500.

It should be emphasized that the dermatoscope itself is not a diagnostic tool but acts to aid decision making in when confronted with a suspicious lesion, allowing the practitioner greater confidence when deciding whether to refer, excise or leave a skin lesion.

The use of the dermatoscope was initially the exclusive realm of the dermatologist, experimental and early work gave rise to extensive descriptions of patterns and features visualised in melanocytic naevi, melanoma and other skin tumours. This then moved to the formalisation of the technique into various algorithms such as pattern analysis [30], the 7-point technique [31], the modified ABCD technique [32] and the Menzies method [33]. Two early meta-analyses of the dermatoscopic technique were published concluding that it increases sensitivity and specificity for the diagnosis of melanoma when compared to the naked eye when in the hands of an experienced clinician [34,35].

In 2004, it was recognised that in order to achieve a decrease in morbidity and mortality, dermoscopy should be a screening test that is available to all practitioners involved in skin screening providing it was accurate, easily to apply and inexpensive. Such a test would have the aim of highlighting suspicious lesions earlier and allow the practitioner to refer patients onto a specialist for further evaluation [36]. Using a randomised controlled trial methodology Westerhoff and colleagues [37] demonstrated it was possible to train a group of non-dermatology expert general practitioners and significantly improve their clinical recognition skills compared with a control group. Argenziano et al [38] reported similar findings with a cohort of 73 primary care physicians. In the UK, courses have been running for a number of years and include a range of health care practitioners. The most recent meta analysis of dermoscopy [36] has encompassed a review of literature including those studies conducted on practitioners with minimal training in the technique and has still concluded a relative diagnostic odds ratio for dermoscopy compared with naked eye examination to be 15.6 (CI 95%; 2.9–83.7, p = 0.01). It therefore seems pertinent to explore the technique as an extension of scope of practice within podiatry. To date the authors are unaware of any published literature documenting its application within this profession.

## The three point technique

The three point technique was developed by Soyer et al [36] who recognised that dermoscopy could be a screening tool for all those involved in skin care. As a result it is a simplified technique to screen suspicious lesions and it particularly useful for the novice. Through the dermatoscope, it assesses individual lesions on three criteria:

(i) Asymmetry of colour and dermatoscopic structures

(ii) Presence of an atypical network

(iii) Presence of blue-white structures or veil

Each criterion, if present scores 1 point. Any lesions scoring two or above should be considered for biopsy and warrant possible excision. A summary of the technique can be found in table 1. A preliminary study of 231 pigmented skin lesions showed that after one hours training six inexperienced dermatologists were able to improve their sensitivity in recognising skin cancer from 69.7% to 96.3% [39]. In a later study with 150 participants, Soyer [36] demonstrated 91% sensitivity, with those in the cohort declaring no experience in dermoscopy still achieving 87% sensitivity for melanoma. Further studies are required to confirm this finding.

**Table 1:** The three point checklist [36]

**Feature**	**Significance**
Asymmetry	Examined in both axes, using the dermatoscope. Colour and structures are assessed. Significant asymmetry of colour or structures within the lesion are recorded as a score of 1.
	
Atypical pigment network	Many naevi have a uniform reticular pattern to the pigment distribution resembling chicken wire or a honeycomb structure with regular brown or black lines. An atypical network is recorded as a score of 1 if the network is irregular in thickness, irregular holes, or irregular colours.
	
Blue structures or blue-white veil	The presence of any blue structure observed including a blue-white veil scores 1.

Any lesion scoring two or more should warrant further investigation – referral/excision

## Dermoscopy and the foot

The dermatoscope has been found useful for the examination of the skin, but the foot has offered a particular challenge to the technique, firstly, because of its thickened acral plantar surface which gives an altered presentation of pigmentation [40] and secondly the nail unit which frequently presents with pigmentation due to a range of causes including haematoma and melanoma. On plantar (and palmar) skin the blue-white veil is rarely observed although asymmetry of colour and shape should still be considered.

**Figure 4:**
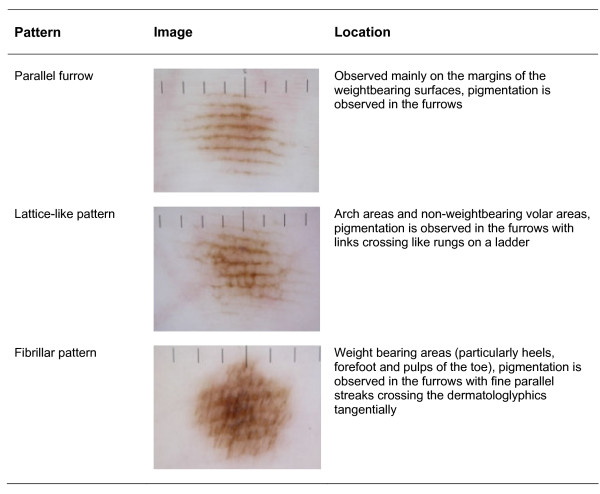
**Dermatoscopic features of benign melanocytic naevi on plantar skin (after Miyazaki et al **[44]**)**.

In addition, other dermatoscopic observations of acral and volar skin have been reported. Saida, Myazaki and colleagues identified 3 specific pigment patterns determined as normal in benign melanocytic naevi of plantar skin parallel furrow, lattice-like and fibrillar pattern [41-44] (figure 4). In each of these the pigment is located in the furrows of the plantar dermatoglyphics. The patterns arise as a reflection of normal melanin columns in the stratum corneum in a vertical (parallel furrow) or slanting fashion [40].

**Figure 5:**
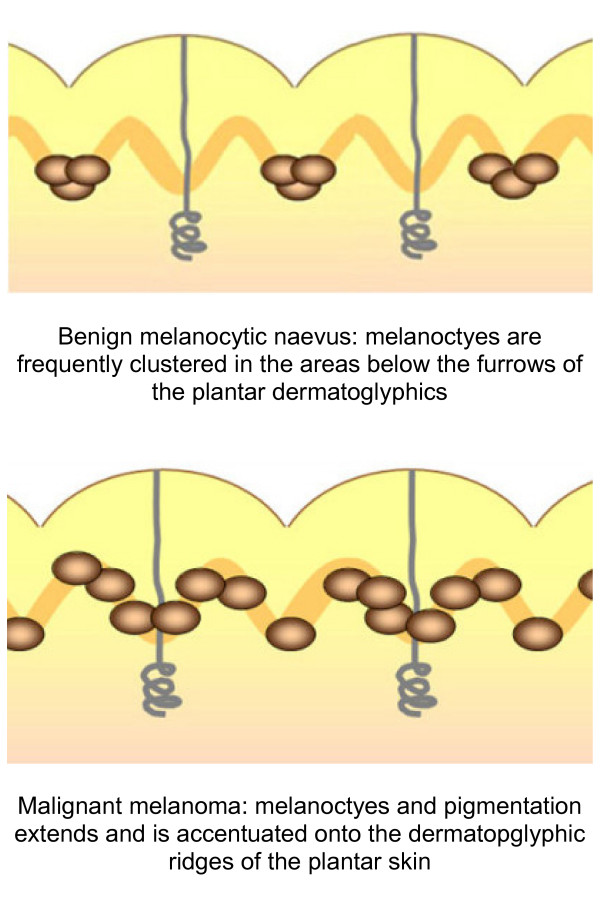
**Melanin distribution patterns on acral skin**.

Malignant melanoma has been shown to exhibit different patterns on the palmar and plantar surfaces. Saida [42] and workers reported, in concordance with the three point algorithm asymmetry and irregular (variegate) colour was a common feature. Furthermore, in malignant melanoma pigmentation is frequently accentuated on the ridges of the dermatoglyphics and not furrows as in benign lesions [45] (Figure 5). To test the hypothesis Saida and colleagues [46] reviewed 712 melanocytic lesions in acral areas, to determine the specificity and sensitivity of these patterns in determining the presence of malignant melanoma. The parallel ridge pattern showed a positive predictive value of 93.7% (the proportion of patients with a proven melanoma who exhibited a parallel ridge pattern) and in benign melanocytic lesions the positive predictive value of the parallel furrow pattern and lattice like pattern were very high at 93.2% and 98.3% respectively (the proportions of patients diagnosed with a benign melanocytic naevus who showed the parallel furrow pattern). The study was carried out on a Japanese cohort although later studies have confirmed the findings in Caucasian populations [47,48].

## Dermoscopy and its potential in assessing nail pigmentation

In addition to the application of the dermatoscope in assessing pigmented plantar lesions, its utility in assessing nail pigmentation has been discussed [49]. A patient presenting with longitudinal melanonychia always presents a diagnostic challenge to Podiatrists due to its various causes such as ethnicity, drugs, trauma and occasionally melanoma. Biopsy of such lesions has the potential to cause permanent scarring to the nail unit. Ronger et al [50] discussed the role of the dermatoscope in nail pigmentation and suggest it as a tool to decide if a nail biopsy should be performed. Subsequent publications have explored this concept further. Braun and colleagues [51] describe the dermatoscopic features of the different causes of melanonychia and have proposed an algorithm. In a similar manner Jellinek [52] suggests it has a role in assessing nails prior to biopsy and again proposes an algorithm. Neither of these have been formally tested to identify their true validity but with time one would expect further development in this area as experience increases.

## Conclusion

Current evidence still demonstrates a rise in the incidence of melanoma, the most lethal form of skin cancer. Without an effective treatment, early detection and excision are vital to improve the prognosis and survival. Lesions located on the foot have been shown to be prone to more diagnostic delays and misdiagnosis compared with tumours elsewhere on the body, subsequently resulting in a poorer prognosis. Dermoscopy is a simple and inexpensive means of visualising pigmented lesions and has been shown to improve diagnostic accuracy. Although originally considered a technique for specialist dermatologist, later developments have suggested that the dermatoscope can be a useful screening tool for health care professionals involved in skin care. On this basis, dermoscopy is potentially a new extension to the scope of practice in Podiatry. In theory, podiatric practice would be well suited for screening pedal lesions. Many patients are routinely seen, particularly the elderly (the age group where most melanoma are observed). The addition of dermoscopy at initial patient assessment may increase not only practitioner awareness but also offer an excellent opportunity to discuss self examination with patients and reinforce the public health message. In its short history the dermatoscope has shown to be effective in highlighting melanoma whilst reducing excisions of benign lesions, but its true capabilities are still being discovered. Continued research, in time, should uncover its true potential.

## Competing interests

The authors declare that they have no competing interests.

## Authors' contributions

IB designed the review, performing the literature search and first drafts of the paper. JB undertook subsequent drafting and the addition of clinical photographs. Both authors read and approved the final manuscript.
